# Imaging of HCC—Current State of the Art

**DOI:** 10.3390/diagnostics5040513

**Published:** 2015-11-27

**Authors:** Christina Schraml, Sascha Kaufmann, Hansjoerg Rempp, Roland Syha, Dominik Ketelsen, Mike Notohamiprodjo, Konstantin Nikolaou

**Affiliations:** Department of Diagnostic and Interventional Radiology, University Hospital Tuebingen, Hoppe-Seyler-Str. 3, Tuebingen 72076, Germany; E-Mails: sascha.kaufmann@med.uni-tuebingen.de (S.K.); h.rempp@yahoo.de (H.R.); roland.syha@med.uni-tuebingen.de (R.S.); dominik.ketelsen@med.uni-tuebingen.de (D.K.); mike.notohamiprodjo@med.uni-tuebingen.de (M.N.); konstantin.nikolaou@med.uni-tuebingen.de (K.N.)

**Keywords:** hepatocellular carcinoma, magnetic resonance imaging, guidelines, diffusion-weighted imaging, dynamic contrast-enhanced magnetic resonance imaging, computed tomography, dual-energy computed tomography, volume perfusion computed tomography, contrast media, hepatocyte specific contrast media

## Abstract

Early diagnosis of hepatocellular carcinoma (HCC) is crucial for optimizing treatment outcome. Ongoing advances are being made in imaging of HCC regarding detection, grading, staging, and also treatment monitoring. This review gives an overview of the current international guidelines for diagnosing HCC and their discrepancies as well as critically summarizes the role of magnetic resonance imaging (MRI) and computed tomography (CT) techniques for imaging in HCC. The diagnostic performance of MRI with nonspecific and hepatobililiary contrast agents and the role of functional imaging with diffusion-weighted imaging will be discussed. On the other hand, CT as a fast, cheap and easily accessible imaging modality plays a major role in the clinical routine work-up of HCC. Technical advances in CT, such as dual energy CT and volume perfusion CT, are currently being explored for improving detection, characterization and staging of HCC with promising results. Cone beam CT can provide a three-dimensional analysis of the liver with tumor and vessel characterization comparable to cross-sectional imaging so that this technique is gaining an increasing role in the peri-procedural imaging of HCC treated with interventional techniques.

## 1. Introduction

Liver cancer is the sixth most common cancer (approximately 750,000 new cases per year) currently representing the third leading cause of cancer-related deaths worldwide [1]. Hepatocellular cancer (HCC) hereby represents over 90% of all primary liver cancers with increasing incidence which is posing a major healthcare problem [1]. This malignancy has an unfavorable prognosis when diagnosed at a late stage because therapeutic approaches are limited. However, effective treatment and long-term survival (50%–70% 5-year survival) are achievable if HCC is diagnosed at an early stage [2]. Thus, early diagnosis of HCC is essential for effective treatment, in order to improve survival of these patients [3].

Risk factors for the development of HCC are chronic viral hepatitis infection, alcoholic and non-alcoholic fatty liver disease, and other types of chronic inflammatory liver diseases [4,5]. Obesity, diabetes and fatty liver disease have also been recognized as risk factors for HCC development, although the underlying mechanisms how these overlapping conditions might contribute to cancer development remain unclear [6,7].

Hepatitis C and B infection are the leading cause of HCC development globally, with hepatitis C being the primary aetiological factor in Western countries [8]. The risk for HCC incidence is depending on the ethology of the hepatitis [9,10]: in hepatitis-C-related cirrhosis the risk lies within the range of 2%–8% per year, while in chronic hepatitis-B-related cirrhosis it is 2.5%, and the risk is reported to be even lower (<2%) in primary biliary and autoimmune cirrhosis [11,12]. The risk for HCC is increasing in case of more than one virus infection and with concomitant alcohol abuse. Moreover, in the general population, the incidence of HCC is higher in patients with HIV infection compared to controls, and HIV appears to be an additive co-factor, increasing the risk of HCC in patients with chronic viral hepatitis [1].

Until the year 2000, the diagnosis of HCC was made primarily by histopathological analysis. In 2001, the European Association for the Study of the Liver (EASL) organized an expert conference in Barcelona, focusing on a clinical diagnosis of HCC, where they agreed to accept non-invasive criteria for diagnosis of HCC, based on a combination of imaging and laboratory findings [13]. To date, HCC is the only tumor in which a fully non-invasive diagnosis is accepted [10].

## 2. Guidelines for Hepatocellular cancer (HCC) Diagnosis—Current Status

Bota *et al.* [10] have given an excellent overview about the current status of guidelines for HCC summarizing similarities and differences between the latest guidelines regarding the non-invasive diagnosis of HCC. They discuss guidelines proposed by the American Association for the Study of Liver Diseases (AASLD), the European Association for the Study of the Liver (EASL) and the Asian Pacific Association for the Study of the Liver (APASL) and the Japanese Society of Hepatology (JSH) [1,14,15,16]. Important differences in non-invasive HCC diagnosis among the Western and Eastern guidelines are reported: Concerning the most appropriate imaging modality for the diagnosis of HCC, different approaches are recommended by the aforementioned four guidelines: the AASLD and EASL guidelines exclusively accept a four-phase contrast-enhanced computed tomography (CT) and/or a dynamic contrast-enhanced magnetic resonance imaging (DCE-MRI), whereas the APASL and Japanese guidelines also allowcontrast-enhanced ultrasound (CEUS). All four guidelines agree on the typical vascular pattern of HCC lesions, being characterized by hyper-enhancement in the arterial phase which is followed by contrast media wash-out in the venous phase. These characteristics (wash-in and wash-out) are caused by the relative difference of contrast media due to differences in arterial and portal venous blood supply of HCC lesions and normal liver tissue. The difference varies in relation to the timing of the injection of contrast agent and the time-point of the acquisition of images [17]. In portal venous and/or late image phases, the portal venous blood contains higher concentrations of contrast media, because the contrast medium recirculates while the bolus in the arterial blood has already left the liver parenchyma. HCC has primarily arterial blood supply, which then has a low concentration of contrast media in the late venous phase. Consecutively, the liver enhances to a greater extent than the HCC in venous or late contrast phases. Thus, the contrast agent is not really “washed out” by the HCC lesion, despite the use of the term [17].

The AASLD and EASL guidelines recommend a diagnostic approach which is primarily based on the assessment of tumor size. The Japanese guidelines and the APASL, however, recommend the application of a diagnostic algorithm which is starting from the analysis of the vascularity pattern, as reflected by hyper- or hypovascular appearance of the tumor in the arterial phase.

As to the choice of contrast media, the AASLD and EASL guidelines advocate the exclusive use of extracellular contrast media in MRI. The APASL guidelines, on the contrary, include superparamagnetic iron oxid which is currently not available or approved for application in all countries. The Japanese guidelines propose the use of liver specific MR contrast media.

### 2.1. HCC Surveillance Program

Surveillance is recommended in all patients with chronic liver disease who are at risk of developing HCC [18]. According to the EASL guidelines there are four categories of adult patients in whom surveillance is recommended: (1) cirrhotic patients (Child-Pugh stage A and B); (2) cirrhotic patients (Child-Pugh stage C awaiting liver transplantation); (3) non-cirrhotic HBV carriers with active hepatitis or family history of HCC and (4) non-cirrhotic patients with chronic hepatitis C and advanced liver fibrosis F3.

The aim of surveillance programs is the early detection of HCC by identifying small lesions in the liver. Small lesions may be HCC, regenerative nodules or dysplastic nodules. The distinction of these three entities is challenging and even complicated by the fact that very early, morphologically identifiable HCC lesions seldom show typical radiological features concerning contrast enhancement patterns. Very early HCC lesions may be hypovascular because of a reduction of their portal venous blood supply, although their arterial vascularization has not yet fully developed [17].

The imaging test most widely used for surveillance is ultrasonography (US) with an acceptable diagnostic accuracy when used as a surveillance test (sensitivity 58%–89%; specificity > 90%) [19,20]. It is a non-invasive method, widely available and associated with moderate costs. Surveillance US, however, should be performed by experienced personnel in all at-risk populations every six months [1,21]. Shorter follow-up is recommended after resection or loco-regional therapies or in case of detection of nodules (<1 cm) in cirrhotic patients. The recommendation to follow small nodules of <1 cm in size by imaging is based on the considerations that accurate needle placement for biopsy is difficult in small nodules, and the majority of such small lesions do not turn out to be HCC [17].

#### 2.1.1. Role of Biomarkers in Surveillance

The clinical role of classical HCC biomarkers such as α-fetoprotein (AFP), lectin-bound AFP (AFP-L3) and protein induced by vitamin K absence or antagonist II (PIVKA-II) has been well documented [15]. However, the sensitivity of these biomarkers in the detection of early HCC or small lesions is limited, and AFP levels may also be elevated in other malignancies (such as cholangiocarcinoma or colon cancer), as well as during flare-ups of chronic viral hepatitis, also in the absence of HCC [10]. To date, the use of AFP or other serological markers for non-invasive HCC diagnosis is not recommended by the European and American Society for the study of liver diseases. The combination of US and AFP has been reported to increase the number of false positives findings as well as costs, so that there is insufficient evidence to support the use of both methods in combination in HCC surveillance programs [22,23,24]. Moreover, the aim of surveillance is the early detection of HCC, which usually does not overproduce AFP [10].

Novel serological biomarkers, however, might be used to improve diagnostic performance and prognostic significance in patients at risk for HCC in the future [5].

#### 2.1.2. Ultrasound in Surveillance

Apart from biomarker analyses, imaging performed by computed tomography (CT), magnetic resonance imaging (MRI) and ultrasonography (US) with or without intravenous contrast administration plays an essential role for early detection of HCC. The European and American expert panels (EASL and AASLD) recommend abdominal ultrasonography every six months for surveillance. The diagnostic performance of US for HCC detection in cirrhotic patients lies within an acceptable range: sensitivity 60%–80% and specificity 45%–94% [25].

### 2.2. Diagnosis of HCC

The correct evaluation of small hepatic nodules in patients at risk for HCC development is of high clinical importance and presents a major challenge for the radiologist.

According to the EASL guidelines, non-invasive criteria for diagnosis of HCC can only be used in cirrhotic patients and should be acquired by a contrast-enhanced four-phase CT scan or a dynamic contrast-enhanced MRI scan. The diagnosis of HCC should be based on the typical hallmarks of HCC (hypervascularity in the arterial phase with washout in the portal venous or delayed phases). The role of contrast-enhanced ultrasound (CEUS) and angiography is discussed controversially; positron emission tomography (PET) imaging is currently not considered sufficiently accurate for early diagnosis of HCC [1], but may have a role for tumor characterization, biopsy guidance and monitoring treatment effects.

**Figure 1:**
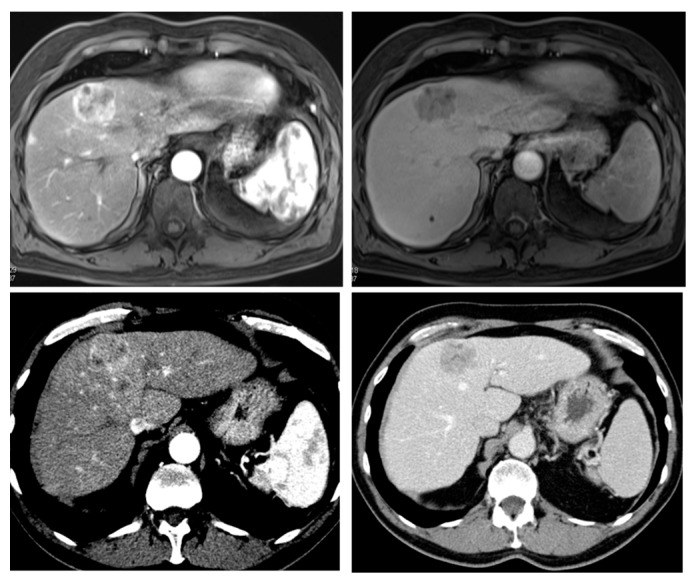
Radiological hallmarks of hepatocellular carcinoma (HCC). Typical vascular pattern of HCC as observed in a 67-year old patient with histology proven HCC. Liver lesion in the right hepatic lobe observed in a cirrhotic patient. The lesion is presenting a typical HCC vascular pattern with arterial hyperenhancement (**left images**) and venous wash-out (**right images**) visible both in magnetic resonance imaging (MRI) (**upper row**) and computed tomography (CT) (**lower row**).

Which imaging technique (CT, MRI or CEUS) should be used for HCC diagnosis? When approaching this question one has to emphasize that currently the HCC radiological hallmark is based on the information about the vascularity pattern of the tumor (Figure 1). This is best obtained in dynamic contrast-enhanced cross-sectional imaging studies, *i.e.*, CT or MRI. The use of US microbubbles, however, is limited in that regard because the microbubbles are confined to the intravascular space as opposed to the extravascular standard contrast agents used in CT or MRI, which are rapidly cleared from the blood pool into the extracellular space [1]. Thus, in CEUS, differentiation of HCC and cholangiocarcinoma (CCC) may be problematic. In their latest version, the EASL recommends the use of the latest generation CT and/or MRI following reported protocols for non-invasive diagnosis of HCC. According to the EASL, perfusion CT or MRI with liver-specific contrast agents may be promising imaging techniques for diagnosing HCC, but have not so far provided sufficient data to support their use as alternate criteria [1].

### 2.3. Contrast Agents

In general, contrast agents used in cross-sectional imaging are divided into two major categories: extracellular intravascular or postvascular phase agents [10]. The postvascular agents, again, can be classified into two groups: The first group consists of contrast agents that are taken-up by hepatocytes and excreted via bile (hepatocyte-specific contrast agents). In this group, there are two MRI contrast agents (Gd-EOB-DTPA (gadolinium ethoxybenzyl diethylenetriamine pentaacetic acid); Primovist^®^, Bayer-Schering, Germany; and Gd-BOPTA; Multihance^®^, Bracco, Italy) that have a regular intravascular phase immediately after injection, but are subsequently extravasating and taken up by hepatocyte and excreted into bile. The second group consists of Sonazoid^®^ (GE Healthcare, UK) and Levovist^®^ (Bayer Schering, Germany) for CEUS and superparamagnetic iron oxide (SPIO) agents for MRI [10].

The AASLD and EASL guidelines accept only image findings provided by extracellular vascular contrast agents [10]. According to the American and European guidelines, only four-phase CT and dynamic contrast MRI is accepted for HCC diagnosis, while the use of contrast-enhanced ultrasound has been skipped, even for second/third-line diagnosis. The Asian Pacific and Japanese guidelines, in contrast, accept contrast-enhanced ultrasound (CEUS) as a diagnostic tool [1,3,10,14,15,16].

### 2.4. HCC Diagnostic Algorithm

**Figure 2:**
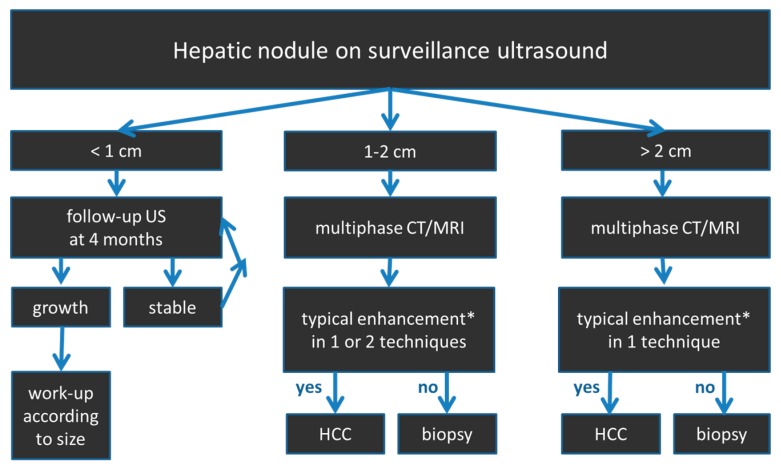
Diagnostic algorithm according to the (European Association for the study of the liver (EASLD) for the evaluation of hepatic nodules in patients at risk for HCC. * typical enhancement: arterial hyperenhancement, portal venous/late phase wash-out.

Guidelines proposed by EASL and AASLD recommend a diagnostic algorithm which is primarily based on the tumor size, whereas the Eastern guidelines follow an algorithm starting from the arterial enhancement characteristics [10]. The EASL guidelines classify lesions into three different categories according to their diameter (<1, 1–2 and >2 cm). A schematic overview of the diagnostic algorithm proposed by the EASL is given in Figure 2.

Lesions <1 cm in diameter are recommended to be followed by US every 4 months (EASL) or 3 months (AASLD), respectively. If the lesion remains constant, follow-up with US should be continued for at least 2 years [1]. For lesions of 1–2 cm in diameter, one or two imaging modalities (contrast-enhanced dynamic CT or MRI scan) are required to establish the diagnosis of HCC depending on the radiological equipment and expertise of the performing imaging center. For lesions >2 cm in diameter, one imaging modality showing the pivotal pattern “arterial hypervascularity and late wash-out” suffices for HCC diagnosis. In case of atypical vascular patterns biopsy is recommended to reach a final diagnosis.

### 2.5. Differentiation of High-Grade Dysplastic Nodules versus HCC

Regarding imaging differentiation between HCC and dysplastic nodules, the AASLD and EASL guidelines currently do not endorse any non-invasive algorithm [1,10,18]. In contrast, the Japanese guidelines already recommend the use of liver specific Gd-EOB-DTPA-MR, because in the hepatocyte specific phase, the vast majority of dysplastic nodules are iso- or hyperintense, whereas most early HCC nodules are hypointense [15].

### 2.6. Differentiating Other Hypervascular Liver Lesions from HCC

There are ***both benign and malignant hypervascular liver tumors*** that need to be differentiated from HCC. Among others, benign hypervascular tumors include arterio-portal shunts, haemangiomas, focal nodular hyperplasia, hepatic adenomas, hyperplastic nodules associated with portal venous abnormalities due to alcohol-induced liver disease or Budd-Chiari syndrome, angiomyolipoma, inflammatory pseudotumors and intrahepatic bile duct adenoma [26].

#### 2.6.1. Benign Hypervascular Liver Lesions

**Figure 3:**
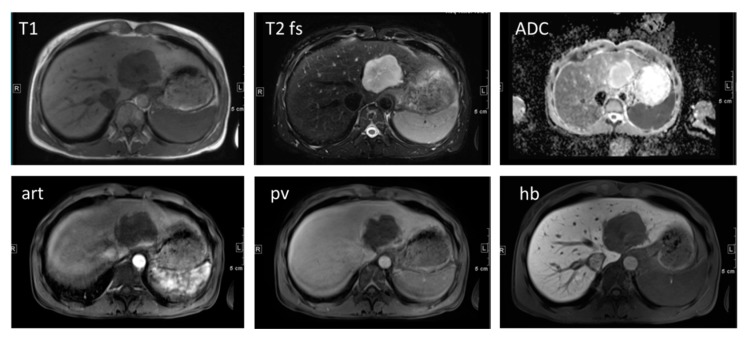
A 47-year old female patient with a large haemangioma in the left liver lobe. Typical image series obtained using hepatocyte specific contrast media is shown. A large lesion is visible in the left liver lobe which presents hypointense in T1, markedly hyperintense in T2 with bright signal in the ADC map. In the contrast-enhanced dynamic series performed after intravenous application of a liver specific contrast agent, the lesions shows centripetal enhancement. In the hepatobiliary phase obtained 20 min after contrast injection, the lesion is hypointense in contrast to the hyperintense liver parenchyma. Haemangiomas do not show the typical pooling appearance in the hepatobiliary phase. Diagnostic confidence can be increased when considering the typically bright signal in T2 and the high ADC values. T1: T1-weighted image; T2: T2-weighted image with fat saturation; ADC: apparent diffusion coefficient map; art: contrast-enhanced image in the arterial phase; pv: contrast-enhanced image in the portal venous phase; hb: contrast-enhanced image in the hepatobiliary phase.

***Haemangioma*** as the most common benign liver tumor is considered to be a developmental malformation that is generally without clinical significance [26]. It is usually hyperechogenic in US, although this is not a specific finding for diagnosis. Dynamic CT and MRI with extracellular contrast media showing the classical fill-in contrast behavior are useful for the diagnosis. However, care should be taken when hepatocyte-specific contrast agents are administered, in order to evaluate hepatic nodules. One needs to be aware that haemangiomas do not show the typical prolonged enhancement in the portal venous and hepatobiliary phase after Gd-EOB-DTPA (Figure 3). This is due to the fact that Gd-EOB-DTPA shows much faster wash-out from the blood pool than do iodine-based contrast agents and Gd-DTPA [26]. Thus, diagnostic confidence for haemangiomas can be improved when considering the T2-weighted images in which typical haemangiomas usually show homogeneous bright signal intensity.

***Hypervascular pseudolesions*** of the liver consist of areas of hyper-enhancement in the arterial phase caused by arterio-portal (AP) shunts. AP shunts usually have a wedge shape and are most commonly observed in the subcapsular region of the liver. A peripheral branch is often seen in the hypervascular region (dot sign), which is a specific finding useful for differentiating hypervascular pseudolesions from hypervascular HCC [26]. Moreover, AP shunts usually show isointensity to the surrounding liver in the hepatobiliary phase of liver-specific contrast agents. Rarely, hypointensity has been reported due to impairment of liver function caused by long-standing AP shunts [27].

**Figure 4:**
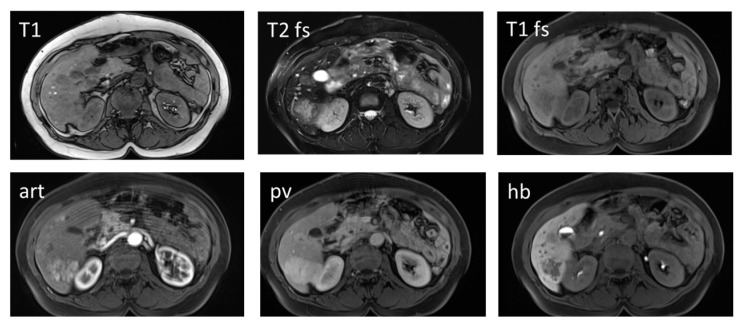
A 47-year old female patient with focal nodular hyperplasia (FNH). Image series obtained before and after administration of hepatocyte specific contrast media is shown. In the right liver, lobe multiple lesions are visible. Lesions present slightly hypointense in T1 and slightly hyperintense in T2. In the contrast-enhanced study using liver specific contrast agent, the lesions show early arterial enhancement which persists in the portal venous phase. In the hepatobiliary phase obtained 20 min after contrast injection, the lesions show persistent contrast enhancement of varying intensity which is most pronounced in the periphery of the largest lesion in segment 6. T1: T1-weighted image in opposed phase; T2: T2-weighted image with fat saturation; T1 fs: T1-weighted image with fat saturation; art: contrast-enhanced image in the arterial phase; pv: contrast-enhanced image in the portal venous phase; hb: contrast-enhanced image in the hepatobiliary phase.

***Focular nodular hyperplasia* (*FNH*)** is a benign hypervascular liver tumor with well-known characteristic image findings such as large feeding artery, small central scar, draining vein and late-phase accumulation of Gd-EOP-DTPA (Figure 4). Moreover, FNH is primarily seen in young women without underlying liver disease, so that the differential to HCC is normally not problematic.

In contrast, ***nodular hyperplastic lesions* (*FNH-like nodules*)** first reported by Gluud *et al.* [28], may be observed in association with alcohol-induced chronic hepatic disease. FNH-like nodules are described as markedly hypervascular nodules of usually less than 2 cm in size which are often difficult to be diagnosed confidently [26]. However, FNH-like nodules are commonly iso- or hyperintense in the hepatobiliary phase in contrast to malignant liver tumors and hepatocellular adenomas, which in the majority of cases show hypointensity in that contrast phase [29]. In case of chronic liver disease, differential diagnosis of FNH-like nodules from HCC may be challenging and accelerated follow-up imaging studies or biopsy may be required.

**Figure 5:**
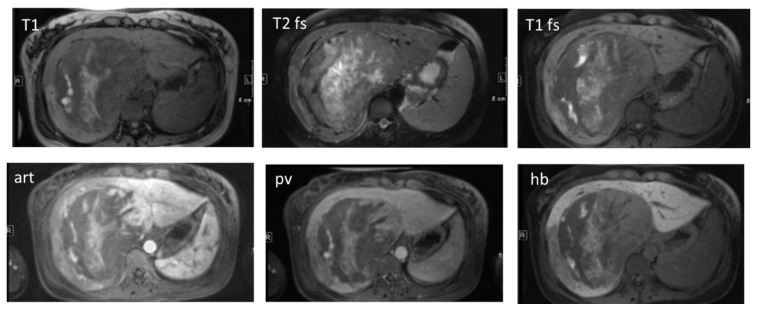
A 24-year old female patient with a large mass in the right liver lobe. Image series obtained before and after administration of hepatocyte specific contrast media is shown. In the right hepatic lobe, a large lesion with heterogeneous signal is visible. Multiple T1-hyperintense structures are present in the lesion representing intralesional hemorrhage. In the contrast-enhanced series, marked arterial enhancement is seen in the solid parts of the lesion. In the hepatobiliary phase, intralesional hemorrhage remains as bright spots while the solid tumor parts in the periphery show moderately hypointense signal in contrast to the brightly enhancing liver parenchyma. The lesion was resected and was proven to be a hepatic adenoma. T1: T1-weighted image opposed phase; T2 fs: T2-weighted image with fat saturation; T1 fs: T1-weighted image with fat saturation; art: contrast-enhanced image in the arterial phase; pv: contrast-enhanced image in the portal venous phase; hb: contrast-enhanced image in the hepatobiliary phase.

Another tumor entity that needs to be differentiated from HCC is the ***hepatocellular adenoma* (*HCA*)**. HCA are principally benign liver neoplasms with specific but variable histopathologic findings and tumor biology [30]. HCA are currently categorized into different subtypes (inflammatory, hepatocyte nuclear factor 1 α mutated, β-catenin-mutated, unclassified), with the inflammatory type being the most common one (40%–50%). Of all HCA, β-catenin-mutated HCA have the highest risk of malignancy and are interpreted as borderline lesions between HCA and HCC [31]. Most patients with HCA are asymptomatic, but in case of large and multiple adenomas hemorrhage may occur (Figure 5).

Typical imaging findings of HCA are homogeneous arterial enhancement and prolonged mild enhancement with smooth margins. HCA usually exhibits hypointensity on hepatobiliary phase images after intravenous (i.v.) administration of Gd*-*EOB*-*DTPA. This finding may be used for distinguishing HCA from FNH but, however, is not useful for the differentiation of HCA from HCC. β-catenin-mutated HCA does not exhibit a specific MR imaging pattern and may mimic HCC [30]. For detailed information, the interested reader may refer to the review focusing on genetics and imaging of HCA by V. Katabathina *et al.* [30].

#### 2.6.2. Malignant Hypervascular Liver Lesions

Malignant hypervascular liver lesions requiring differentiation from HCC comprise cholangiocarcinoma, cholangiolocellular carcinoma, mixed tumor containing HCC and cholangiocarcinoma and hepatic metastases [26]. Cross-sectional image findings are helpful to diagnose these lesions.

***Cholangiocarcinoma* (*CC*)** present with arterial hypervascularity in the majority of cases. The most helpful criterion for differentiation from HCC is the characteristic feature of late enhancement in CC and CCC lesions which is caused by intratoumoral fibrosis. Besides, there exists a mixed type of liver cancer which is composed of HCC and CC stem cells. This mixed liver cancer type presents with varying imaging features and is often difficult to diagnose against HCC [26].

***Hypervascular liver metastases*** in generally do not lead to relevant diagnostic problems regarding their differentiation against HCC, especially when they are observed in otherwise healthy livers and when the primary cancer is already known. In unclear cases, biopsy may be required.

### 2.7. Treatment Response Evaluation in HCC Patients

Current cross-sectional imaging modalities provide reliable and reproducible anatomical data for the assessment of tumor burden changes. In view of the increasing use of novel targeted therapies, the measurement of anatomical size changes may be less useful for response evaluation under therapy, because under these therapies, changes in size generally appear later than biological changes [32]. As a consequence, there is an increasing demand to monitor treatment response at an early phase and to detect functional and biological therapy-induced changes in HCC lesions by measuring functional parameters as reflected by diffusion and perfusion properties or tumor metabolism by PET [32].

The awareness of the importance of adding functional information to the treatment response evaluation is reflected in the modified RECIST (mRECIST) criteria. In mRECIST, viable tumor is measured, defined as areas with uptake of contrast agent in the arterial phase. In view of the broad availability of various imaging techniques, it is challenging to determine the most appropriate image criteria that can provide most significant surrogate parameters for treatment response in HCC with adequate cost efficiency. To date, the new mRECIST criteria are recommended in daily routine for treatment response assessment, because they include not only tumor diameters but also lesion viability [32].

The timing of treatment response evaluation recommended in the literature varies depending on the treatment approach: After resection, ablation or transarterial chemoembolization (TACE), the initial response evaluation should be performed four weeks after treatment. After liver transplantation, the initial imaging control should be scheduled at 3 months. Patients with more advanced stages of HCC who are treated with TACE or systemic agents (e.g., sorafenib) should be screened clinically for signs of liver decompensation and should be evaluated with cross-sectional imaging for tumor progression using dynamic CT or MRI every two months, to direct treatment decisions [32]. Serum tumor markers may be useful in treatment response assessment if their levels were high at diagnosis and decreased after treatment. Nevertheless, tumor markers cannot replace imaging modalities in most cases [32].

### 2.8. Challenges of the Diagnostic (Imaging) Algorithms

When adhering to these diagnostic algorithms in patients with increased risk for HCC, there are three particular scenarios that should once again be looked at separately: very early HCC, hypovascular HCC and cholangiocarcinoma. Very early HCC are usually less than 2 cm in size and do not necessarily already show the pivotal vascularity pattern of HCC [17]. However, in histopathologic analyses, they already exhibit morphological alterations indicating malignancy such as stromal invasion [33]. These lesions contain fat and therefore may be hyperechogenic in US and hypodense in CT [17]. Being hypovascular in both arterial and late phase, the lesion does not yet meet the criteria for establishing an HCC diagnosis non-invasively by classical imaging criteria. Thus, these lesions should be biopsied and analyzed by an experienced pathologist. In addition to very early HCC, there is a small number of larger, later-stage HCCs which may also present as hypovascular lesion [17]. These lesions should also be biopsied accordingly. Third, the differentiation of HCC *versus* intrahepatic cholangiocarcinoma is important because treatment and prognosis differ. Intrahepatic cholangiocarcinoma is a tumor that may also be found in cirrhotic livers. It shows hyperenhancement (mostly peripheral) on the arterial phase which is corresponding to the viable cellular tumor. In the later phases, centripetal filling and contrast retention is observed which is explained by fibrous tissue and necrosis due to the presence of loose connective tissue and extracellular matrix [34,35,36]. The absence of wash-out in a lesion in a cirrhotic liver should therefore always raise the suspicion of ICC [37].

### 2.9. Liver Imaging Reporting and Data System (LI-RADS) Categorization

The Liver Imaging and Reporting and Data System (LI-RADS) is a system of standardized terminology and criteria for hepatic CT and MRI studies [38] which was developed by internationally recognized medical and surgical experts with reference to opinions and evidence obtained from the literature [39,40]. LI-RADS aims at helping radiologists to improve the consistency and clarity in interpretation and reporting of CT and MRI examinations of the liver [38]. LI-RADS currently only applies to patients with cirrhosis or otherwise high risk of HCC. LI-RADS provides a diagnostic algorithm based on a lexicon of precisely defined terms and imaging features, to help radiologists in assigning a relative probability for HCC to untreated observations visualized in the cirrhotic liver [39]. Based on this algorithm, categories from LR-1 (definitely benign) to LR-5 (definite HCC) are assigned indicating the likelihood of benignity or HCC [39]. In the 2014 version of LI-RADS, hepatobiliary contrast agents have been included in the diagnostic algorithm [41]. The updated version of LI-RADS v2014 is available for free on the ACR homepage [38]. In addition to imaging criteria, LI-RADS also outlines technical requirements for CT and MRI studies in order to achieve a more standardized imaging technique among different institutions [39]. The use of LI-RADS is not only useful for radiologists when categorizing observations but might also help referring physicians understand radiologic reports [42]. The validation of LI-RADS in large histology-proven prospective studies will be a major issue in the next years.

## 3. Magnetic Resonance Imaging (MRI)

MRI is the imaging modality of choice in the diagnosis of HCC. In the following paragraph we will focus on three particular aspects of MRI in the diagnosis of HCC: hepatocyte specific MR contrast agents, the role of diffusion-weighted imaging and the potential of fast dynamic sequences for optimized temporal resolution in dynamic contrast-enhanced MRI of the liver.

### 3.1. Hepatocyte Specific MRI Contrast Agents

Two classes of MR contrast agents can be considered as hepatocyte- or liver-specific: first reticuloendothelial cell-specific agents such as superparamagnetic iron oxide (SPIO) and second gadolinium-based hepatocyte-specific agents. SPIO is phagocyted by hepatic Kupffer cells. Malignant lesions tend to contain less Kupffer cells and therefore appear less hypointense than healthy liver tissue in SPIO imaging [43,44]. Thus, SPIO enables functional liver imaging; however, well-differentiated HCC retain functional Kupffer cells and may therefore not be detected using SPIO [35,45].

**Figure 6:**
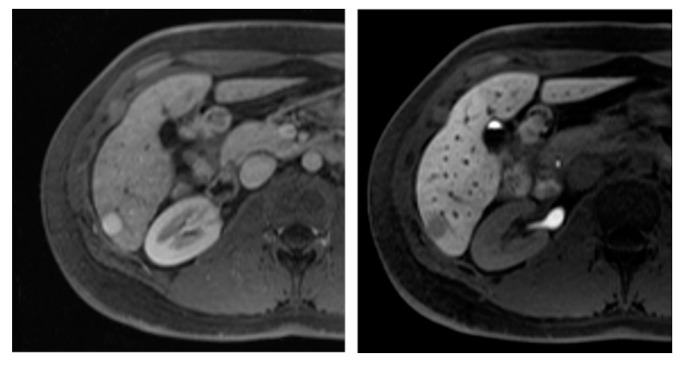
Typical MR imaging features of HCC using hepatocyte specific contrast media. Axial MR images of the liver obtained after intravenous administration of a hepatocyte specific contrast agent in arterial (left) and hepatobiliary (right) phase obtained 20 min after i.v. contrast application. In segment 6, a hypervascular lesion is observed. In the hepatobiliary phase image, the lesion is hypointense in relation to the surrounding liver due to reduced uptake of contrast agent due to loss of functioning hepatocytes in the dedifferentiated HCC lesion.

Gadobenate dimeglumine (Gd-BOPTA, Multihance^®^, Bracco, Milan, Italy) and gadoxetate acid (Gd-EOB-DTPA, gadolinium ethoxybenzyl diethylene-triamine pentaacetic acid, Primovist^®^ or Eovist^®^, Bayer Schering Pharma, Berlin, Germany) are gadolinium-based MR contrast agents with liver-specific uptake behavior [46]. In contrast to non-specific, extracellular MR contrast agents, these are taken up by the hepatocytes and excreted to the bile ducts by specific protein transporters. They therefore enable to obtain functional information during the hepatobiliary examination phase, as the T1 signal of the Gd-containing hepatocytes is increased. Low differentiated hepatocellular carcinomas, metastases from extrahepatic primaries as well as haemangiomas and other lesions without specific uptake remain with low T1 signal and can therefore be differentiated throughout the hepatobiliary phase. Typical image findings observed in HCC using a hepatocyte-specific MRI contrast agent can be seen in Figure 6.

During early multiphasic dynamic examination, liver-specific contrast agents show imaging dynamics similar to extracellular contrast agents. This is especially useful for the detection of hepatocellular carcinoma, where early arterial enhancement followed by wash-out is considered the pivotal imaging characteristic. As the uptake of contrast media by hepatocytes immediately starts when the contrast agent is within the vascular and extracellular space, the signal of healthy liver parenchyma starts to increase early and an overlap between the delayed phase of the dynamic examination and the beginning hepatobiliary phase should be taken into consideration. Especially using Gd-EOB-DTPA, the classical wash-out can often not be detected due to this effect [47].

The uptake behavior of the liver-specific contrast agents differs. Gadoxetate acid is taken up by specific transporter molecules into the hepatocyte due to its lipophilic moiety. About 50% of the injected dose is excreted to the bile ducts; therefore, the recommended dose is four times less than that of extracellular agents [48]. Using gadoxetate acid, hepatobiliary phase imaging can be acquired 10–20 min after the administration of contrast agent [49]. Using gadobenate dimeglumine, hepatobiliary phase occurs 40 min after the administration, as only 2%–4% of the injected volume is taken up by the hepatocytes, while the rest is cleared by the kidneys [50].

One has to be aware that the hepatobiliary phase may be delayed in cirrhotic patients due to a reduced liver function [51]. Criteria for the diagnostic validity of hepatobiliary phase are the hypointense appearance of the vessels towards the liver parenchyma and hyperintense bile ducts resulting of biliary contrast agent excretion. While in healthy liver, gadoxetate acid and gadobenate dimeglumine were reported to lead to similar enhancement, gadoxetate acid achieves better liver enhancement in cirrhotic patients [52]. In non-cirrhotic livers, there is no evidence for a difference between gadoxetate acid and gadobenate dimeglumine in the diagnostic performance [53]; therefore gadoxetate acid may be preferred due to its early uptake and shorter examination time.

Diagnostic accuracy of hepatocyte-specific agents is higher than that of SPIO-enhanced MRI for small, moderately- to well-differentiated HCC [54]. Compared to MDCT, diagnostic accuracy of hepatocyte-specific agents is superior especially in case of small lesions (<1.5 cm) [55,56]. Hepatocyte-specific agents are especially useful for the differentiation of HCC from regenerative or dysplastic nodules as well as for the differentiation from focal nodular hyperplasia (FNH) and liver adenoma [57,58,59].

If hepatobiliary phase imaging is desired using gadoxetate acid, both axial T2w and DWI can be performed after the acquisition of the contrast enhanced late dynamic phase in order to shorten examination time [60]. If an additional T2w MRCP is necessary, it should be performed before the administration of gadoxetate acid [60].

It is worth noting that liver-specific contrast agents may be limited for the detection of well-differentiated HCC (with remaining functional hepatocytes) and for the detection of HCC in cirrhotic liver (with generally decreased liver function and imaging contrast). Moreover, the differentiation of HCC from other solid liver lesions such as haemangiomas or from post-therapeutic changes such as chemotherapy-induced focal hepatopathy can be challenging and should be done using complementary information of dynamic examination and DWI [61].

### 3.2. Diffusion-Weighted MR Imaging

**Figure 7:**
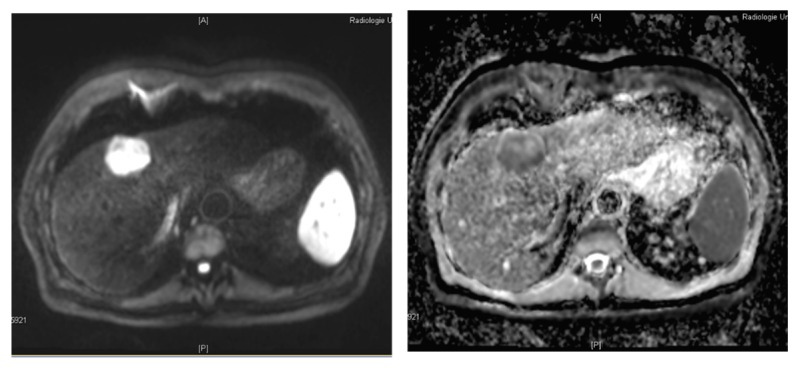
Diffusion-weighted imaging in a 67-year old patient with histology proven HCC. Liver lesion in the right hepatic lobe observed in a cirrhotic patient (same patient as in Figure 1). The HCC lesion is presenting with high signal in the diffusion-weighted image (left) with high *b*-value (*b* = 800 s/mm^2^) and shows reduced ADC when compared to the surrounding liver parenchyma (right).

Diffusion-weighted imaging (DWI) is a functional MR imaging technique that relies on the Brownian motion of water molecules and uses the differences in mobility of water protons for contrast generation in the images. The mobility of the water molecules is inversely influenced by the cell density and the integrity of the cell membranes [62]. Thus, a characterization of the microstructure of the tissue is possible. The diffusivity of the tissue can be quantified by calculating the apparent diffusion coefficient (ADC). The lower the ADC, the higher is the diffusion restriction in the tissue. Cirrhotic liver has an altered architecture with multiple regenerative nodules and bridging fibrous septa [63]. A small proportion of these nodules may undergo malignant transformation. With advancing dedifferentiation, cellular and structural atypia of the nodule is increasing which—at a microscopic level—is characterized by an increase in cellular density, nuclear-to-cytoplasmic ratio and thickening of the cellular plates [64]. The named changes contribute to impairment of the water molecule mobility which leads to an increased diffusion restriction and thus a reduced ADC. As a consequence, the diffusivity of a cirrhotic nodule may serve as a surrogate parameter for its malignant potential and degree of dedifferentiation [63]. This enables great diagnostic potential of DWI for diagnosing HCC which has been investigated in various studies in the recent years [63,65,66,67,68,69,70,71,72,73,74,75]. Diffusion-weighted images obtained in a histology-proven HCC lesion are shown in Figure 7.

Interestingly, the low *b*-value images (in the range <100 s/mm^2^) have great potential for diagnosing HCC with reported sensitivities for HCC of 73% to >90% [72,74]. They have been reported to enhance liver-to-lesion contrast because the T2 signal in small intrahepatic blood vessels and bile ducts is suppressed while signal-to-noise ratio is still kept at an acceptable high level in comparison to high *b*-value images. High *b*-value images, in contrast, provide high specificity for differentiation of HCC *versus* benign cirrhotic lesions [76]. Apart from the chosen *b*-values, the degree of fibrosis markedly influences the conspicuity of nodules in cirrhotic livers because fibrosis also restricts the mobility of water molecules which increases the signal of liver parenchyma in diffusion-weighted images.

To date, there is no consensus regarding the optimal *b*-value for diagnosing HCC in cirrhosis [63]. Moreover, the optimal analytic model for ADC calculation in HCC diagnosis has not yet been defined. According to the theory of intravoxel incoherent motion (IVIM) [77], the motion of water protons measured in DWI consists of two components: the fast perfusion fraction and the slow diffusion component. These two components can be separated by using a bi-exponential fit which might be helpful when assessing highly vascularized tumors such as HCC. Woo *et al.* [78], investigated 42 surgically proven HCC with IVIM and reported that IVIM-derived diffusion parameters of HCC showed significantly better diagnostic performance than ADC values for the differentiation of high-grade *versus* low-grade HCC and also observed significant correlation of the perfusion fraction with arterial enhancement. Recently, diffusion kurtosis imaging (DKI) has been tested for hepatic applications [79,80]. In DKI, the deviation of the apparent diffusivity in the tissue from Gaussian behavior is measured with a dimensionless metric termed the “excess kurtosis” that is representing the microstructural environment of the tissue [79]. Goshima *et al.* [79], who compared DKI with conventional DWI for response assessment in hypervascular HCC, suggest that DKI could be a new option for treatment response evaluation in HCC.

The dedifferentiation of cirrhotic nodules to HCC (hepatocarcinogenesis) is considered a continuous process and, thus, the visibility of hepatic lesions in DWI is dependent on the actual lesion status along this pathway [63]. In a study performed by Muhi *et al.* [76] in 2009, more than 90% of moderately and poorly differentiated HCC were visible in diffusion-weighted imaging (*b*-values 500 and 1000 s/mm^2^), while approximately 50% of well-differentiated HCC and all dysplastic nodules were not visible.

With DWI being a functional imaging technique that provides quantitative parameters, the technique has great potential for treatment response monitoring which also holds true for the different treatment options chosen in HCC patients including locoregional ablative methods such as TACE, radiofrequency ablation and radioembolization but also systemic treatment with anti-angiogenic drugs [81,82,83,84,85,86]. Significant increase in ADC with reduced contrast enhancement has been observed in HCC treated with TACE [82,85]. Interestingly, the time interval between treatment and imaging seems to play an important role for the presence of ADC changes: According to the study by Kamel *et al.* [82], the increase in ADC was most distinct 7-14 days after TACE but insignificant 1 day and four weeks after TACE.

In contrast to the quantitative ADC approach, the visual analysis of diffusion-weighted images has been reported to be less sensitive for detecting local residual/recurrent tumor post TACE [87]. When using absolute quantification of diffusivity by means of ADC for HCC assessment one has to be aware of the limitations of the technique starting with limited inter- and intra-observer reliability of ROI analyses in the ADC map and lack of standardization regarding *b*-values or fitting algorithm (mono- *versus* bi-exponential) [77]. Moreover, the presence of blood products may lead to variable T2 prolongation and water diffusivity with considerable impact on signal characteristics in DWI and also on the value in the corresponding ADC map [88].

For a more detailed summary of the role of DWI in diagnosis of HCC the interested reader may refer to the review by K.S. Lim focusing on the role of DWI MRI of HCC in cirrhosis published in 2014 [63].

### 3.3. Dynamic Contrast Enhanced MRI

Dynamic contrast enhanced MRI analyses the dynamic contrast alteration caused by the passage of a gadolinium containing contrast agent bolus through the organ of interest [89,90,91,92]. As HCC lesions are typically well vascularized, they form ideal targets for assessment with DCE-MRI. The signal dynamics are modeled as a function of time, so that either semiquantitative parameters such as, “time to peak (TTP)” or “area under the curve (AUC)” [93], or quantitative parameters modeling a tissue’s pharmacokinetics can be derived [90,94]. Due to the non-linear relation of gadolinium concentration to measureable signal only small amounts of contrast agents (<50% body weight adapted dose) are required [95]. Up to date, there is still no consensus regarding the optimal acquisition protocol for DCE-MRI of the liver. Principally, all studies exploit strongly T1-weighted pulse sequences [89,90,91,92,96,97]. Inflow-artefacts can be suppressed using inversion- or saturation techniques [98]. Current 3D-sequences allow the entire organ of interest to be covered in a sufficiently high temporal resolution for pharmacological modeling [99].

Quantitative parameters based on pharmacokinetic two-compartment models are supposed to be independent of the contrast agent delivery rate, the contrast agent concentration and the scanning hardware [96]. However, there is a known dependency of these parameters on the pharmacokinetic model applied. Models primarily investigating HCC, which is supplied by the arterial vascular system, exploit a single arterial input function, whereas investigation of liver parenchyma or metastases necessitates a dual-input arterial and portal-venous input function [94]. Generally, the following parameters can be derived from most pharmacokinetic 2-compartment-models [96]:

(1) *k*^trans^—the exchange constant defining the flux from the blood vessels into tissue;

(2) *k*_ep_—the exchange constant, defining the reflux from tissue into the vascular system; and

(3) *V*_E_—the volume fraction of the extravascular extracellular space (EES).

If hepatocyte-specific contrast agents are used for DCE-MRI, the pharmacokinetic model has to consider the hepatocyte uptake of the contrast agent [100].

The parameters derived from DCE-MRI analysis can be used for differential diagnosis of intrahepatic lesions. HCC lesions typically exhibit a stronger perfusion and faster mean transit time compared to colorectal metastases [89]. Furthermore, DCE-MRI holds value for therapy-monitoring of HCC. Responders and non-responders of thalidomide-therapy can be distinguished based on significant differences for the semiquantitative parameters maximum enhancement and maximum enhancement slope [92]. Therapy with antiangiogenetic tyrosine-kinase-inhibitors causes a reduction of *k*^trans^ and *k*_ep_ with an inverse correlation between decreasing *k*^trans^ and therapy response [101,102]. Clinical outcome can be predicted by analyzing pharmacokinetics before and after the first therapy cycle [90,91]. The degree of *k*^trans^ decline also shows correlation to time-to-progression and expression of vascular endothelial growth factor (VEGF), as well as to markers of anti-hypoxia [101].

Reproducibility of *k*^trans^ is good to moderate, with a variance of 15%–40%, so that changes of more than 40% can be considered as a therapy effect [103]. However, there is still a lack of multicenter studies and a consensus regarding acquisition technique, postprocessing and cut-off values [96].

## 4. Computed Tomography (CT)

Cross sectional, dynamic and multiphase contrast-enhanced computed tomography (CT) is often the first diagnostic imaging technique to diagnose HCC, and besides, MRI represents the standard imaging method. For daily clinical routine, CT is the best available technique to diagnose HCC, and in contrast to CEUS it is examiner-independent. Thus, CT allows a fast, reproducible and inexpensive examination. Especially for patients with reduced general state of health and restricted compliance, CT offers an adequate examination in contrast to MRI or CEUS, which are more dependent on the compliance of the patient. Dynamic multidetector-row computed tomography (MDCT) has achieved increased spatial and temporal resolutions due to increasing gantry rotation speed and rising number of detector rows. Thus, high quality 3D images can be reconstructed from isotropic voxel imaging data by using multiplanar reconstruction, volume rendering and maximum intensity projection techniques. 3D images have been reported to be useful for preoperative evaluation of liver anatomy [104].

CT imaging has developed from a 2-dimensional imaging (2D) to a 3-dimensional (3D) imaging modality, and further to so-called 4-dimensional (4D) imaging, which allows the analysis of the dynamics of contrast media distribution over time. For CT, strictly extracellular iodine-based, contrast agents are used. The contrast media kinetics are analyzed by multiphasic acquisitions, generally obtained before, during and after contrast agent administration. This is extremely important for HCC and its differential because diagnosis is based on changes in blood supply and hemodynamics [105]. Classical multiphase MDCT of the liver consist of non-contrast, late arterial, portal venous, as well as equilibrium phase imaging [106]. The non-contrast phase is useful to detect hyperattenuation due to haemorrhage or hyperattenuating embolic agents like lipiodol before contrast administration, thus avoiding misinterpretation of arterial-phase hyperenhancement [107]. The late arterial phase which is characterized by full enhancement of the hepatic artery and beginning enhancement of the portal vein is useful to detect hypervascular HCC [108]. The portal venous phase which is characterized by enhancement of hepatic veins as well as portal veins and the equilibrium phase imaging are useful for the differential diagnosis of HCC, especially differentiation from arterio-portalvenous shunting [106]. The majority of HCCs show washout of contrast medium in these phases [109]. But not all HCCs reveal this [110]. Early HCCs and dysplastic nodules are often iso- or hypo-vascularized in dynamic CT with different enhancement patterns compared to hypervascularized advanced HCCs [105,111,112,113,114]. Early HCC can show iso-attenuation throughout all phases of dynamic CT [115]. Early HCCs receive blood supply from hepatic artery as well as from portal vein, but the number of intratoumoral portal veins supplying these lesions is less than one third of that in the surrounding liver tissue [116,117]. In the largest whole-liver-explant study Peterson *et al.* [118], evaluated patients who underwent tri-phasic CT imaging comparing results with those obtained by histology and reported a prospective sensitivity of tri-phasic CT for detection of HCC, judged on a nodule-by-nodule basis, of only 37%. Lim *et al.* [110], detected 71% of HCC and 39% of dysplastic nodules in patients who underwent multiphase-CT and subsequently liver transplantation. Rode *et al.* [13], detected 2% of DN and 54% of HCC using tri-phasic CT in cirrhotic patients prior to liver transplantation. A meta-analysis comparing the diagnostic accuracy of CT and MR imaging for HCC in patients with chronic liver disease shows that MRI is more sensitive than CT for diagnosing HCCs (79% *vs.* 72%) [68]. Another meta-analysis reported a sensitivity of 81% for CT as compared to 91% for MRI whereas the specificity of CT was 93% compared to 95% for MRI [119]. Tsurusaki *et al.* [120] compared the sensitivity for preoperative detection of HCCs and found a higher sensitivity for gadoxetic acid-enhanced MRI than for multiphasic MDCT imaging.

### 4.1. Advances in CT Imaging

Several technical advances and CT imaging techniques such as dual-energy CT (DECT) and volume perfusion CT (VPCT) are currently being explored for improving detection, characterization and staging of HCC.

### 4.2. Dual-Energy CT (DECT)

Dual-energy CT is technique, which enables differentiation of materials, and tissues based on their CT density values, using two different x-ray energy and absorption spectra. With DECT, non-contrast phase imaging can be reconstructed from contrast-enhanced data applying a virtual non-contrast (VNC) algorithm acquired by tin-filter-enhanced, dual-source, dual-energy computed tomography (DECT) [121,122]. Wang *et al.* [123] described that quantitative analysis of the DECT spectral curve in the portal venous phase can be used to determine whether tumors are benign or malignant.

Dai *et al.* [124], investigated the value of volumetric iodine-uptake (VIU) changes as measured by dual-energy CT (DECT) for treatment response evaluation after sorafenib in comparison to AASLD criteria (American Association for the Study of Liver Diseases). They could show that DECT can evaluate the disease control consistent with the current standard AASLD. Lee *et al.* [125], described that color-coded iodine CT is comparable to conventional liver CT protocols in demonstrating viable HCCs after TACE, while it allows a reduction in radiation dose. Despite promising first results of DECT, currently there are no large studies demonstrating benefit of dual-energy CT over conventional MDCT.

### 4.3. Volume-Perfusion CT (VPCT)

Increasing arterialization is a characteristic of HCC. In the past, CT hepatic arteriography (CTHA) and CT during arterial portography (CTPA) were performed, to evaluate the hemodynamics of hepatocellular lesions [126]. Ohki *et al.* [127], described that CTHA/CTAP may detect recurrent lesions earlier. The combination of CTHA and CTAP is recommended to improve the detection sensitivity of small hypervascular HCC nodules [128]. The use of CTHA/CTAP has been recommended to improve detection of HCCs [129,130]. However, the use of CTAP and CTHA is invasive, costly and often requires a multivessel approach.

Volume perfusion CT (VPCT) is performed by the acquisition of serial images after a bolus administration of 30–40 mL of iodinated contrast medium, enabling detailed, non-invasive analysis of liver hemodynamics [131,132]. VPCT equipped with dedicated software allows separately quantification of the dual arterial and portal-venous supply of liver parenchyma and tumor. Fischer *et al.* [133], described that visual analysis of perfusion maps shows good sensitivity for detection of HCC and that additional assessment of anatomical arterial MIPs further improves detection rates of HCC. Perfusion CT enables assessment of HCC vascularity, by adding quantitative information about the presence of arterial vessels within neoplastic [134,135]. VPCT provides assessment of perfusion changes in HCC after chemoembolization, which can be used to evaluate the therapeutic response of chemoembolization for hepatocellular carcinoma [90]. In advanced HCC, VPCT is a more sensitive image biomarker for monitoring early antiangiogenic treatment effects as well as in predicting outcome at the end of treatment and progression-free survival as compared with RECIST and tumor density [136]. In patients with HCC treated with TACE, perfusion CT provides measurement of flow parameters related to residual arterial structures in viable tumor, thus helping in the assessment of therapeutic response [137]. Radiation exposure from perfusion CT could be lowered with iterative reconstruction techniques and provides similar quality at a lower dose compared with the usual filtered back-projection algorithm [138]. Although currently not yet included in standard diagnostic guidelines, perfusion CT may offer additional information regarding liver hemodynamics and blood flow directed toward tumors in the liver [139].

### 4.4. Cone Beam Computed Tomography in Peri-Procedural Imaging of HCC

**Figure 8:**
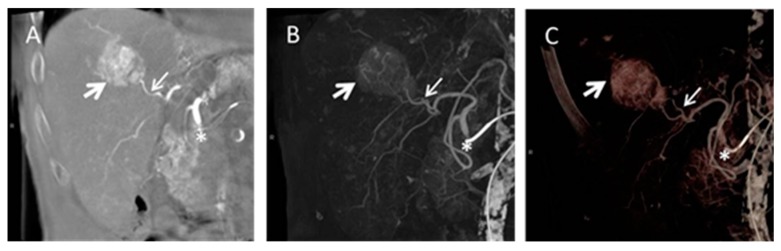
Cone beam computed tomography (CB CT) gained during transarterial chemoembolization (TACE). Coronal multiplanar reconstruction (**A**), coronal maximum intensity projection; (**B**) and volume rendering technique; (**C**) are presented. The tumor volume is marked by the big white arrow and the tumor feeding vessel is marked by a smaller white arrow. The asterisk marks the catheter position in the common hepatic artery.

Cone beam computed tomography (CB CT) is increasingly used for peri-procedural assessment of TACE and selective internal radiation therapy (SIRT) in HCC as it shows advantages in immediate pre-interventional treatment planning [140,141,142,143]. In especial, detection of smaller or less vascularised liver tumors is reported to be limited using conventional angiography due to inhomogeneous perfusion in cirrhotic liver tissue [144,145,146]. The main advantage of CB CT as part of an angiographic suite consists of intra-procedural acquisition of 3D volumes of interest and further post-processing including maximum intensity projections (MIP) and multiplanar reconstructions (MPR) comparable to conventional cross-sectional imaging, as well as acquisition of unenhanced and contrast enhanced images of liver parenchyma [140] (Figure 8).

**Figure 9:**
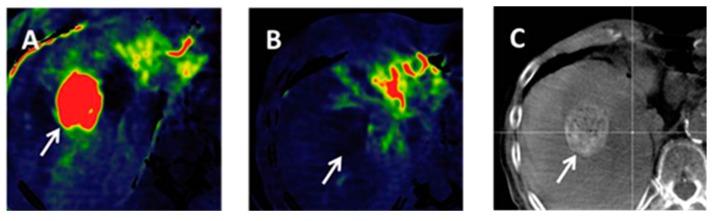
Parenchymal blood volume (PBV) in a HCC lesion before (**A**) and after (**B**) drug eluting bead (DEB) TACE. The tumor volume is marked by the white arrow in all three images; (**B**) shows disappearance of PBV after treatment; (**C**) demonstrates contrast media staining after delivery of beads typically for DEB TACE.

Regarding TACE CB CT shows advantages in detection of HCC lesions and tumor vessels [147]. Kakeda *et al*. [148] reported that CB CT in pre-treatment evaluation of catheter positioning added useful information in 81% of the cases. Tognolini *et al.* [149] furthermore described a substantial change of treatment procedure in up to 30% of patients and a detection rate of supplementary lesions of 15%. Challenging techniques are dual phase CB CT and parenchymal blood volume (PBV). Dual phase CB CT allows acquisition of different contrast phases which might have further impact on lesion detectability [150]. PBV requires acquisition of unenhanced and contrast enhanced CB CT. Advantage of PBV is acquisition of three dimensional (3D) cross-sectional images including non-contrast and contrast-enhanced anatomical information as well as perfusion based data [151]. PBV is based on an application used for detection of cerebral blood volume [152] and first experiences in evaluation of HCC have been published recently [153,154,155]. PBV values gained during CB CT acquisition are comparable to perfusion characteristics obtained by volume perfusion CT and might be of further interest in patient pre-selection, pre-treatment planning, and treatment evaluation of TACE in HCC (Figure 9).

Considering SIRT CB CT has potential in detection of extrahepatic shunting and non-perfusion of target segments during SIRT. Van den Hoven *et al.* [143], reported a negative predictive value of 95% for extrahepatic shunting and 83% for lack of target segment perfusion.

## 5. Future Work

Imaging plays a pivotal role in diagnosis and management of patients with hepatocellular carcinoma. Although ultrasound is currently the main surveillance imaging tool for HCC [156], dynamic cross-sectional CT and MR imaging studies will remain the primary imaging techniques applied for diagnosis and staging of HCC in the next years. This is supported by the ongoing technical advances on the field of CT and MR technology concerning reduction of radiation exposure, optimization of tissue characterization, development of targeted contrast agents and introduction of postprocessing software used for evaluation of perfusion studies but also for texture analysis. The prospective evaluation of the role of sophisticated MR techniques in the diagnosis of HCC, such as contrast-enhanced MR perfusion imaging, arterial spin labeling, MR elastography, intralesional iron and fat quantification should be engaged in future studies.

LI-RADS is a promising approach for the unification of HCC categorization systems in CT and MRI imaging studies which is aimed at more consistent image interpretation and reporting of hepatic imaging studies in patients at increased risk for HCC. An important step in this direction is establishment of common language and definitions, which will facilitate the interdisciplinary dialogue in clinical routine and research [40]. As the LI-RADS approach is currently only applicable to untreated observations, the evaluation of standardized evaluation criteria for treated HCC should be promoted in the future.

## 6. Conclusions

Diagnosing HCC at an early stage is crucial for optimizing treatment outcome of patients at risk for HCC. This underlines the importance of adequate imaging studies, applying US, MRI and CT. The Western guidelines proposed by the AASLD and the EASL recommend a diagnostic algorithm starting from the tumor size with follow-up in nodules <1 cm, and one imaging modality with typical contrast behavior in nodules >2 cm. Depending on the expertise of the imaging center, one or two imaging modalities with typical contrast characteristics are required for a definite HCC diagnosis in nodules between 1 and 2 cm in diameter. The radiological hallmark of HCC is the classical pattern of early arterial enhancement, followed by wash-out in the venous phase.

Ongoing advances are made in imaging of HCC regarding detection, classification, grading and staging and also with respect to treatment monitoring possibilities. The potential of MRI with fast dynamic contrast-enhanced imaging and the use of hepatocyte specific contrast agents is increasingly being recognized in HCC diagnosis although currently not yet implemented in the Western guidelines. Functional imaging techniques such as diffusion-weighted MR imaging provide both qualitative and quantitative tools both for grading of HCC but also for treatment response evaluation. Computed tomography as a fast, cheap and easily accessible imaging modality has a major role in the clinical routine work-up of HCC. Technical advances in CT such as DECT and VPCT are currently being explored for improving detection, characterization and staging of HCC, with promising results. CB CT can provide a three-dimensional analysis of the liver with tumor and vessel characterization comparable to cross-sectional imaging, so that this technique is gaining an increasing role in the peri-procedural imaging of HCC treated with TACE and SIRT.
